# The impact of multiple sclerosis onset symptom on cardiac repolarization

**DOI:** 10.1002/brb3.742

**Published:** 2017-06-05

**Authors:** Alma Mikkola, Aku Ojanen, Juha E. K. Hartikainen, Anne M. Remes, Sakari Simula

**Affiliations:** ^1^ Department of Neurology Kuopio University Hospital Kuopio Finland; ^2^ Institute of Clinical Medicine – Neurology University of Eastern Finland Kuopio Finland; ^3^ Department of Clinical Physiology and Nuclear Medicine Mikkeli Central Hospital Mikkeli Finland; ^4^ Heart Center Kuopio University Hospital Kuopio Finland; ^5^ Institute of Clinical Medicine – Medicine University of Eastern Finland Kuopio Finland; ^6^ Medical Research Center Oulu University Hospital Oulu Finland; ^7^ Research Unit of Clinical Neuroscience, Neurology University of Oulu Oulu Finland; ^8^ Department of Neurology Mikkeli Central Hospital Mikkeli Finland

**Keywords:** autonomic nervous system, cardiac repolarization, multiple sclerosis, onset symptom

## Abstract

**Introduction:**

Multiple sclerosis is associated with prolonged cardiac repolarization but the underlying physiology has remained unknown. In this study, we compared cardiac repolarization during the relapsing‐remitting multiple sclerosis (RRMS) disease course in patients with motor and sensory onset symptom.

**Methods:**

Twenty‐five RRMS patients with motor and 33 RRMS patients with sensory onset symptom having 12‐lead electrocardiogram (ECG) recorded at the time of the first demyelinating event (ECG1) as well as at the later disease course (ECG2) were identified from the patient records. The average time interval between ECG1 and ECG2 was 8.6 ± 5.9 y. Heart rate‐corrected QT intervals reflecting cardiac repolarization were calculated by Bazett (QTcBaz), Fridericia (QTcFri), and Karjalainen (QTcKar) formulas.

**Results:**

Heart rate‐corrected QT intervals as well as heart rate were similar in patients with motor and sensory onset symptom in ECG1. However, QTcBaz (*p* = .002), QTcFri (*p* = .019), and QTcKar (*p* = .026) were longer and heart rate was higher (*p* = .035) in patients with motor than sensory onset symptom in ECG2. Correspondingly, QTcBaz (*p* = .002), QTcFri (*p* = .033), and QTcKar (*p* = .043) prolonged and heart rate tended to increase (*p* = .060) during the disease course only in the patients with motor onset symptom.

**Conclusions:**

Cardiac repolarization prolonged and heart rate increased during the disease course in RRMS patients with motor but not with sensory onset symptom. This suggests different traits in RRMS according to its initial manifestation and also association of motor onset symptom with more unfavorable cardiovascular prognostic determinants.

## 1 INTRODUCTION

Multiple sclerosis (MS) debilitates a multitude of central nervous system processes, including motor, sensory, and cognitive domains (Wynia, Middel, Van Dijk, De Keyser, & Reijneveld, 2008). In addition, up to two‐thirds of the patients with MS demonstrate cardiac autonomic dysfunction (Acevedo, Nava, Arriada, Violante, & Corona, 2000; Nasseri, Tenvoorde, Adèr, Uitdehaag, & Polman, 1998; Racosta, Kimpinski, Morrow, & Kremenchutzky, 2015).

The central autonomic network (CAN) is a connectome matrix between insular cortex, amygdala, hypothalamus, periaqueductal gray matter, nucleus tractus solitarius, and ventrolateral medulla (Cersosimo & Benarroch, 2013). In addition to other autonomic functions, CAN modulates cardiac repolarization and heart rate (Magnano, Holleran, Ramakrishnan, Reiffel, & Bloomfield, 2002). Cardiac repolarization is mirrored by heart rate‐corrected QT (QTc) interval in an electrocardiogram (ECG) (Postema & Wilde, 2014). Previously, QTc interval has been reported to be longer in patients with MS than in healthy subjects (Drouin, Nataf, Lande, & Louboutin, 1998; de Seze et al., 2000).

Prolonged QTc interval is a predictor for unfavorable cardiovascular events (Oikarinen et al., 2004; Porthan et al., 2009) even in subjects without cardiac disease (Schouten et al., 1991; Siscovick et al., 1996). In patients with MS, cardiovascular mortality has been reported to be higher than in general population (Brønnum‐Hansen, Koch‐Henriksen, & Stenager, 2004; Manouchehrinia, Tanasescu, Tench, & Constantinescu, 2016). On the other hand, the domain of the onset symptom has been demonstrated to predict MS‐related (Sumelahti, Tienari, Wikström, Salminen, & Hakama, 2002) as well as all‐cause (Midgard, Albrektsen, Riise, Kvåle, & Nyland, 1995) mortality in patients with MS. Whether the domain of the onset symptom in relapsing‐remitting MS (RRMS) has an impact on QTc interval during the disease course, however, is not known.

In this study we compared QTc interval and its dynamics during the disease course between RRMS patients demonstrating their onset symptom either in motor or in sensory domain.

## 2 MATERIAL AND METHODS

We carried out an analysis of data collected retrospectively from the patient records of Mikkeli Central Hospital and Kuopio University Hospital. All the patients with the diagnosis of RRMS and onset symptom either in motor or sensory domain were initially screened from our patient records (*n* = 183). Only patients in sinus rhythm, with clinically definitive RRMS (according to the McDonald 2010 criteria (Polman et al., 2011)) and without any cardiovascular disease influencing cardiac repolarization at the time of onset symptom were included (*n* = 168). Finally, 58 of these patients had baseline 12‐lead electrocardiogram (ECG) recorded at the time of the first exacerbation (ECG1) as well as the latest nonacute 12‐lead ECG recorded at the later course of RRMS (ECG2) for comparison, and were included in the analyses.

All the onset symptoms affecting pyramidal tract were defined as motor. On the other hand, the onset symptom was defined as sensory if optic neuritis or somatosensory exacerbation (paresthesia or neuropathic pain) occurred without any motor defect. All the onset symptoms lasted over 24 hr and were considered suggestive of the first demyelinating event by clinicians.

Kuopio University Hospital Research Ethics Committee approved the study protocol and the research was carried out in accordance with the Declaration of Helsinki (2008) of the World Medical Association. According to the recommendations of local ethics committee, the authorization for using a register data was obtained from the record controller. Informed consent was not required, because of the register‐based nature of the study.

### 2.1 Patients

The onset symptom was motor in 43% (*n* = 25) and sensory in 57% (*n* = 33) of 58 patients. Of the 33 patients with sensory onset symptom of RRMS, 55% (*n* = 18) had isolated optic neuritis and 45% (*n* = 15) had somatosensory onset symptom.

The data on concomitant medication and diseases were obtained from the patient records. At the time of ECG1, 20% (5/25) of the patients with motor and 12% (4/33) of the patients with sensory onset symptom had any concomitant disease and the concomitant disease burden was found similar between the groups (*p* = .479). The prevalence of asthma (12% vs. 6%; *p* = .643), rheumatoid arthritis (8% vs. 0%; *p* = .181), osteoporosis (4% vs. 0%; *p* = .431), depression (0% vs. 6%; *p* = .501), and schizophrenia (4% vs. 0%; *p* = .431) showed no statistical difference between patients with motor and sensory onset symptom, respectively.

At the time of ECG2, 48% (12/25) of the patients with motor and 33% (11/33) of the patients with sensory onset symptom had any concomitant disease (*p* = .258). Namely, diabetes (0% vs. 6%; *p* = .501), hypertension (8% vs. 12%; *p* = .690), coronary artery disease (0% vs. 3%; *p* = 1.000), asthma (12% vs. 6%; *p* = .643), rheumatoid arthritis (8% vs. 0%; *p* = .181), osteoporosis (4% vs. 0%; *p* = .431), depression (12% vs. 6%; *p* = .643), bipolar disorder (0% vs. 3%; *p* = 1.000), schizophrenia (4% vs. 0%; *p* = .431), malignancy (0% vs. 3%; *p* = 1.000), epilepsy (4% vs. 3%; *p* = 1.000), and hypothyroidism (4% vs. 0%; *p* = .431) were recorded, but the prevalence of these in patients with motor and sensory onset symptom did not differ, respectively.

In case of disease‐modifying treatment (DMT), patients were either on interferon beta compounds, glatiramer acetate, or natalizumab. None of the patients were on oral DMTs. Concomitant medication was found similar between the patients with motor and sensory onset symptom both at the time of ECG1 and ECG2 (Table 1).

**Table 1:** Medication at the time of onset symptom of relapsing‐remitting multiple sclerosis either in motor or in sensory domain (ECG1) and later disease course (ECG2)

	*ECG1*			*ECG2*		
	Motor (*n* = 25)	Sensory (*n* = 33)	*p*‐value	Motor (*n* = 25)	Sensory (*n* = 33)	*p*‐value
Cardiovascular medication						
ACE inhibitors	0	0		0	1 (3)	1.000
ATII receptor blockers	0	0		1 (4)	3 (9)	.627
β‐blocking agents	0	1 (3)	1.000	1 (4)	4 (12)	.378
Ca‐channel blocking agents	0	0		1 (4)	1 (3)	1.000
Diuretics	0	0		0	2 (6)	.501
Nitrates	0	0		0	1 (3)	1.000
Anti‐cholinergics	0	5 (15)	.063	2 (8)	7 (21)	.275
Anti‐depressants	3 (12)	7 (21)	.490	6 (24)	11 (33)	.439
Anti‐epileptics	2 (8)	0	.181	5 (20)	7 (21)	.910
Anti‐inflammatorics	11 (44)	15 (45)	.921	9 (36)	20 (61)	.063
Anti‐psychotics	1 (4)	0	.431	1 (4)	1 (3)	1.000
Muscle relaxants	2 (8)	0	.181	5 (20)	5 (15)	.731

ACE, angiotensin‐converting enzyme; ATII, angiotensin II. Values are number (%) of patients.

### 2.2 Assessment of cardiac repolarization in electrocardiogram

The 12‐lead ECGs were recorded in supine position as a part of routine practice. QT interval was assessed by automatic analysis and was defined as the interval between the start of QRS complex and the end of *T* wave. All the analyses were manually confirmed. QT interval is influenced by heart rate and, therefore, needs to be adjusted accordingly. In this study, the calculation of heart‐rate corrected QT intervals was performed using the Bazett (QTcBaz) (Bazett, 1920), the Fridericia (QTcFri) (Fridericia, 1920), and the Karjalainen (QTcKar) (Karjalainen, Viitasalo, Mänttäri, & Manninen, 1994) formulas.

### 2.3 Statistical analysis

Kolmogorov‐Smirnov test was applied to verify the normal distribution of the variables. Comparisons of continuous variables were performed using the independent samples *t* test and comparison of categorical variables using the Chi‐square and Fisher's Exact test between the patients with motor and sensory onset symptom as well as between the patients with optic neuritis and somatosensory onset symptom. A paired samples *t* test was used for within group comparisons. All statistical analyses were performed using IBM SPSS Statistics for Macintosh (Version 22.0; Released 2013; Armonk, NY, USA). Results are expressed as mean ± standard deviation (*SD*), unless otherwise indicated. A *p*‐value ≤.05 was considered statistically significant.

## 3 RESULTS

The RRMS patients with motor and sensory onset symptom were similar with respect to age, gender, systolic and diastolic blood pressure, as well as IgG‐index indicating the ratio between [cerebrospinal fluid immunoglobulin G vs. albumin] and [serum immunoglobulin G vs. albumin], at the time of ECG1 (Table 2). The average time interval between ECG1 and ECG2 was 8.6 ± 5.9 years, with no difference between the patients with motor (8.3 ± 6.9 years) and sensory (8.9 ± 5.0 years) onset symptom (*p* = .704).

**Table 2:** Clinical characteristics of patients with motor and sensory onset symptom of relapsing‐remitting multiple sclerosis

	Motor (*n* = 25)	Sensory (*n* = 33)	*p*‐value
Age (years)	35 ± 12	35 ± 12	.964
Female gender (%)	17 (68)	15 (45)	.087
Li‐Leuk (×10^6^/L)	7.4 ± 9.2	12.0 ± 19.0	.313
IgG‐index	1.12 ± 0.46	1.07 ± 0.52	.720
Na (mmol/L)	139 ± 2	140 ± 3	.567
K (mmol/L)	3.9 ± 0.3	4.0 ± 0.3	.132
sBP (mmHg)	133 ± 16	136 ± 23	.621
dBP (mmHg)	80 ± 11	82 ± 11	.610

Li‐Leuk, leukocyte concentration in cerebrospinal fluid; IgG‐index, the ratio between [cerebrospinal fluid immunoglobulin G vs. albumin] and [serum immunoglobulin G vs. albumin]; Na, plasma sodium concentration; K, plasma potassium concentration; sBP, systolic blood pressure; dBP, diastolic blood pressure. Values are mean ± *SD* or number (%).

During follow‐up, 81% (47/58) of the patients were on any DMT. DMTs were used in 72% (18/25) of the patients with motor and in 88% (29/33) of the patients with sensory onset symptom, with no difference between the groups (*p* = .179).

### 3.1 Cardiac repolarization at the early course of RRMS

Similar heart rate (74 ± 13 bpm vs. 70 ± 14 bpm; *p* = .238) and QT interval (371 ± 35 ms vs. 379 ± 28 ms; *p* = .342) were found in ECG1 in patients with motor and sensory onset symptom, respectively.

In ECG1, heart rate corrected QTcBaz (408 ± 17 ms vs. 406 ± 19 ms; *p* = .549), QTcFri (395 ± 19 ms vs. 396 ± 14 ms; *p* = .838), and QTcKar (398 ± 17 ms vs. 398 ± 14 ms; *p* = .957) were similar in patients with motor and sensory onset symptom, respectively.

### 3.2 Cardiac repolarization during the course of RRMS

The patients with motor onset symptom showed significantly longer QTcBaz (424 ± 25 ms vs. 408 ± 17 ms; *p* = .002), QTcFri (406 ± 18 ms vs. 395 ± 19 ms; *p* = .033), as well as QTcKar (407 ± 16 ms vs. 398 ± 17 ms; *p* = .043) in ECG2 than in ECG1, respectively. Uncorrected QT interval was similar between ECG2 and ECG1 (371 ± 24 vs. 371 ± 35 ms; *p* = .981), whereas heart rate had a trend to be higher in ECG2 than in ECG1 (79 ± 13 bpm vs. 74 ± 13 bpm; *p* = .060), respectively.

The patients with sensory onset symptom, on the other hand, showed no change in QTcBaz (406 ± 19 ms vs. 406 ± 19 ms; *p* = .981), QTcFri (395 ± 15 ms vs. 396 ± 14 ms; *p* = .675), or in QTcKar (397 ± 15 ms vs. 398 ± 14 ms; *p* = .735) between ECG2 and ECG1, respectively. Correspondingly, in patients with sensory onset symptom, no change was found in heart rate (70 ± 14 bpm vs. 72 ± 14 bpm; *p* = .550) or in QT interval (379 ± 28 ms vs. 376 ± 31 ms; *p* = .503) between ECG1 and ECG2, respectively.

**Figure 1:**
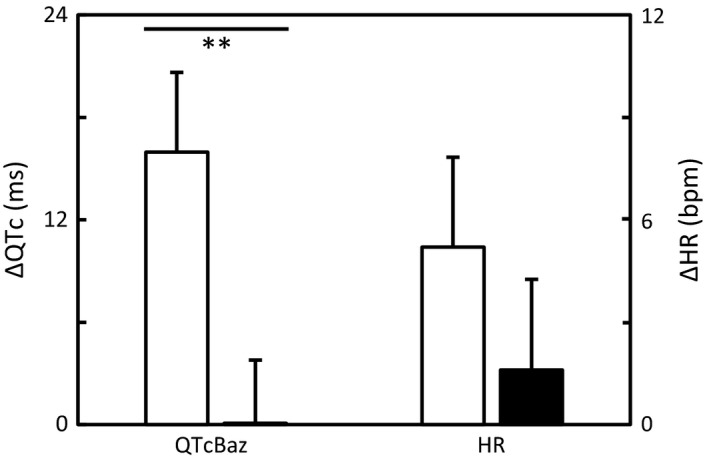
Changes in heart rate‐corrected QT interval (∆QTc) assessed by the Bazett formula (QTcBaz) and in heart rate (∆HR) between the onset symptom (ECG1) and later disease course (ECG2) in patients with motor (white bar) and sensory (black bar) onset symptom of RRMS. Values are mean ± *SEM*. Significance: **p < .01

The prolongation in heart rate‐corrected QT interval was more distinct in patients with motor than sensory onset symptom as calculated in absolute units in QTcBaz (16.0 ± 23.4 ms vs. 0.1 ± 21.2 ms; *p* = .009) (Figure 1), in QTcFri (10.3 ± 22.8 ms vs. −1.2 ± 16.5 ms; *p* = .030), and in QTcKar (8.7 ± 20.3 ms vs. −0.9 ± 15.8 ms; *p* = .047) as well as in relative units in QTcBaz (4.0 ± 5.7% vs. 0.2 ± 5.3%; *p* = .012), in QTcFri (2.8 ± 5.9% vs. −0.2 ± 4.2%; *p* = .027) and in QTcKar (2.3 ± 5.1% vs. −0.2 ± 4.0%; *p* = .044), respectively.

### 3.3 Cardiac repolarization at the later course of RRMS

At the time of ECG2, heart rate was higher in patients with motor (79 ± 13 bpm) than in patients with sensory (72 ± 14 bpm; *p* = .035) onset symptom. Despite this, uncorrected QT interval was similar between the patients with motor (371 ± 24 ms) and sensory (376 ± 31 ms; *p* = .564) onset symptom in ECG2.

**Figure 2:**
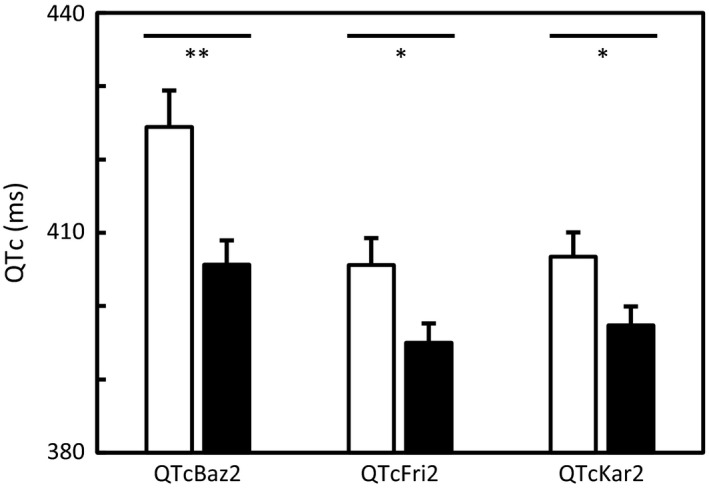
Heart rate‐corrected QT intervals at the later disease course (ECG2) assessed by Bazett (QTcBaz2), Fridericia (QTcFri2), and Karjalainen (QTcKar2) formulas in patients with motor (white bar) and sensory (black bar) onset symptom of RRMS. Values are mean ± *SEM*. Significances: *p ≤ .05 and **p < .01.

Significantly longer QTcBaz (424 ± 25 ms vs. 406 ± 19 ms; *p* = .002), QTcFri (406 ± 18 ms vs. 395 ± 15 ms; *p* = .019), and QTcKar (407 ± 16 ms vs. 397 ± 15 ms; *p* = .026) were observed in ECG2 in patients with motor onset symptom as compared to patients with sensory onset symptom, respectively (Figure 2).

None of the patients had QTc >450 ms in ECG1 as calculated by Bazett, Fridericia, or Karjalainen formula. In ECG2, however, QTcBaz values >450 ms were found in five (20%) patients with motor but only in 1 (3%) patient with sensory onset symptom with borderline statistical significance (*p* = .075) for difference.

The patients with isolated optic neuritis and those with somatosensory onset symptom demonstrated comparable demographics and heart rate‐corrected QT intervals both at the time of ECG1 and ECG2 (Table 3). We found no change between ECG1 and ECG2 in heart rate (*p* = .893; *p* = .473) or in QTcBaz (*p* = .749; *p* = .713), QTcFri (*p* = .542; *p* = .989), or QTcKar (*p* = .462; *p* = .870) in patients with isolated optic neuritis or in patients with somatosensory onset symptom, respectively.

**Table 3:** Clinical characteristics of patients with optic neuritis and somatosensory onset symptom of relapsing‐remitting multiple sclerosis

	Optic neuritis (*n* = 18)	Somatosensory (*n* = 15)	*p*‐value
Age (years)	33 ± 13	37 ± 10	.307
Female gender (%)	7 (39)	8 (53)	.407
Li‐Leuk (×10^6^/L)	6.9 ± 8.5	17.9 ± 25.9	.150
IgG‐index	0.96 ± 0.39	1.20 ± 0.63	.210
Na (mmol/L)	140 ± 3	139 ± 2	.687
K (mmol/L)	4.0 ± 0.2	4.0 ± 0.3	.960
sBP (mmHg)	137 ± 28	134 ± 15	.713
dBP (mmHg)	78 ± 12	86 ± 10	.103
HR1 (bpm)	69 ± 15	71 ± 12	.791
HR2 (bpm)	70 ± 14	74 ± 15	.470
QTcBaz1 (ms)	408 ± 20	403 ± 17	.514
QTcFri1 (ms)	399 ± 15	393 ± 13	.247
QTcKar1 (ms)	401 ± 15	395 ± 13	.169
QTcBaz2 (ms)	406 ± 20	405 ± 18	.928
QTcFri2 (ms)	397 ± 17	393 ± 13	.483
QTcKar2 (ms)	399 ± 16	395 ± 13	.481
Follow‐up (y)	8.0 ± 3.4	9.9 ± 6.4	.305

HR, heart rate; QTcBaz, heart rate‐corrected QT interval according to Bazett formula; QTcFri, hear rate‐corrected QT interval according to Fridericia formula; QTcKar, heart rate‐corrected QT interval according to Karjalainen formula; Follow‐up, time interval between ECG1 (1) and ECG2 (2). Other abbreviations are explained in the footnote of the Table 2. Values are mean ± *SD* or number (%).

## 4 DISCUSSION

In this study, we demonstrated that cardiac repolarization prolonged during the disease course in patients with motor but not in patients with sensory onset symptom of RRMS. Furthermore, patients with sensory onset symptom either in optic neuritis or somatosensory domain were found to have similar characteristics of cardiac repolarization and heart rate throughout the disease course.

Patients with motor and sensory onset symptom did not differ with respect to cardiac repolarization at the time of the first demyelinating event (ECG1). However, heart rate‐corrected QT interval prolonged during the disease course particularly in patients with motor onset symptom. The motor onset symptom of RRMS has conventionally been associated with worse prognosis and more pronounced disability accumulation (Bsteh et al., 2016; Damasceno, Von Glehn, Brandão, Damasceno, & Cendes, 2013; Eriksson, Andersen, & Runmarker, 2003). Our present finding demonstrates that the RRMS patients with motor onset symptom are more prone also to the prolongation of cardiac repolarization during the disease course. This finding enhances the concept that different traits in RRMS are determined already at the early phase of the disease.

The first demyelinating event in optic neuritis (Confavreux, Vukusic, & Adeleine, 2003; Tintore et al., 2015) or somatosensory (Eriksson et al., 2003) domain are both considered as the signs of more favorable prognosis in RRMS. Correspondingly, patients with sensory onset symptom as a whole or in optic neuritis or somatosensory subgroup demonstrated comparable QTc interval features and heart rate at the time of the first demyelinating event (ECG1) as well as at the later disease course (ECG2). Accordingly, the conventional signs for favorable outcome at the early phase of the disease associates also with lower impact of RRMS on cardiac repolarization.

At the later phase of the disease, heart rate was found higher in patients with motor than in patients with sensory onset symptom of RRMS. Clinical characteristics including age and gender, comorbidities, medication, and the duration of follow‐up were similar between these two groups and thus, may not explain the finding. Higher heart rate in combination with longer heart rate‐corrected QT interval even strengthens the concept of diverse effects of motor and sensory onset symptom on cardiac autonomic regulation during the long‐term RRMS disease course. Lower cardiovascular fitness is associated with higher resting heart rate (Tulppo, Mäkikallio, Seppänen, Laukkanen, & Huikuri, 1998), and this should be borne in mind while interpreting our results. However, on our opinion, differences in heart rate may not significantly confound the interpretation of our findings as heart rate‐corrected QT interval namely allows comparison of cardiac repolarization between different heart rate.

Prolongation in heart rate‐corrected QT interval has previously been shown to be associated with spinal cord atrophy secondary to axonal loss in MS patients (de Seze et al., 2000). Indeed, preganglionic autonomic neurons convey in the lateral horn, whereas motor and sensory neurons locate in their own tracks within spinal cord (Bican, Minagar, & Pruitt, 2013). Possible differences in the devastation in the CAN and spinal autonomic pathways during the motor and sensory RRMS onset disease courses may be one explanation for our findings. Differences in disease activity during the years of RRMS may also have an impact on our findings. However, the differences in disease activity are possibly determined already at initial disease trait as previously demonstrated (Bsteh et al., 2016).

Previously, cardiovascular sympathetic dysfunction has been reported in patients with clinically isolated syndrome (CIS) (Crnošija et al., 2016). However, the entity of CIS is different from RRMS as one‐third of the patients are suggested to remain monophasic without further disease activity after the first demyelinating event (Brownlee & Miller, 2014). In addition, QTc interval is influenced by complex interaction between sympathetic and parasympathetic nervous system at the level of CAN, peripheral nervous system and intrinsic cardiac nervous system (Cersosimo & Benarroch, 2013; Shen & Zipes, 2014) and thus, cannot be considered as a pure marker of cardiovascular sympathetic function.

Evaluation of ECG parameters, an average of 8 years apart, is exceptional in real‐life RRMS research. As Bazett, Fridericia, and Karjalainen formulas yielded comparable results, the methodological concerns related to the QT interval adjustment remain low. On the other hand, the relatively small sample size and the retrospective nature of the study are acknowledged as factors possibly limiting the interpretation of the results.

## 5 CONCLUSIONS

Disease traits are, in terms of cardiac autonomic regulation, different after motor and sensory onset symptom of RRMS. Cardiac repolarization prolongs and heart rate tends to increase after motor onset symptom but remain stable after sensory onset symptom. This finding enhances the understanding of different traits in RRMS. In addition, the importance of cardiovascular evaluation in RRMS patients is highlighted, as prolongation of cardiac repolarization has clinical implications not only in drug safety issues but also in risk stratification for subsequent cardiovascular events.

## CONFLICT OF INTEREST

The authors declare that there is no conflict of interest.
